# Tau-Induced Pathology in Epilepsy and Dementia: Notions from Patients and Animal Models

**DOI:** 10.3390/ijms19041092

**Published:** 2018-04-05

**Authors:** Marina P. Sánchez, Ana M. García-Cabrero, Gentzane Sánchez-Elexpuru, Daniel F. Burgos, José M. Serratosa

**Affiliations:** 1 Laboratory of Neurology, IIS (Instituto Investigación Sanitaria/Health Research Institute)-Jiménez Díaz Foundation, UAM (Universidad Autonoma de Madrid/Autonomous University of Madrid) and Biomedical Research Network Center on Rare Diseases (CIBERER), 28045 Madrid, Spain; amgarcia@cnb.csic.es (A.M.G.-C.); gselexpuru@gmail.com (G.S.-E.); daniel.fburgos@quironsalud.es (D.F.B.); joseserratosa@me.com (J.M.S.); 2 Department of Immunology and Oncology and Protein Tools Unit, Biotechnology National Center (CNB/CSIC), 28049 Madrid, Spain

**Keywords:** dementia, epilepsy, tau, mouse models, cognitive impairment, Alzheimer’s disease, seizures, neuronal excitability, FTDP-17 mouse model

## Abstract

Patients with dementia present epilepsy more frequently than the general population. Seizures are more common in patients with Alzheimer’s disease (AD), dementia with Lewy bodies (LBD), frontotemporal dementia (FTD) and progressive supranuclear palsy (PSP) than in other dementias. Missense mutations in the microtubule associated protein tau (MAPT) gene have been found to cause familial FTD and PSP, while the P301S mutation in MAPT has been associated with early-onset fast progressive dementia and the presence of seizures. Brains of patients with AD, LBD, FTD and PSP show hyperphosphorylated tau aggregates, amyloid-β plaques and neuropil threads. Increasing evidence suggests the existence of overlapping mechanisms related to the generation of network hyperexcitability and cognitive decline. Neuronal overexpression of tau with various mutations found in FTD with parkinsonism-linked to chromosome 17 (FTDP-17) in mice produces epileptic activity. On the other hand, the use of certain antiepileptic drugs in animal models with AD prevents cognitive impairment. Further efforts should be made to search for plausible common targets for both conditions. Moreover, attempts should also be made to evaluate the use of drugs targeting tau and amyloid-β as suitable pharmacological interventions in epileptic disorders. The diagnosis of dementia and epilepsy in early stages of those diseases may be helpful for the initiation of treatments that could prevent the generation of epileptic activity and cognitive deterioration.

## 1. Epilepsy in Dementia

Epileptic seizures are more common in patients with dementia than in the general elderly population [1,2]. Seizures in these patients may result from alterations in inhibitory-excitatory systems, although the precise cause of their generation is still not entirely understood. The most common cause of dementia is Alzheimer’s disease (AD), accounting for 60 to 70% of total cases of dementia (Available online: http://www.who.int/mediacentre/factsheets/fs362/es/). Epileptic seizures are more frequent in patients with AD than in patients with other dementias [3,4,5,6,7,8,9,10], and especially affect early-onset forms and more advanced AD. Incidence of seizures and myoclonus is also higher in patients with dementia with Lewy bodies (DLB) and frontotemporal dementia (FTD) than in the normal population. The seizure incidence rates, as compared to normal populations, have been estimated to be around 10-fold in AD and DLB, and 6-fold in FTD [11]. Moreover, epileptic seizures have also been reported in PSP [12], and in Down syndrome (DS) [8], and they have been associated with the presence of dementia. Myoclonic seizures, usually considered as a consequence of hyperexcitability within the sensory-motor cortex, are also frequent in patients with AD [13,14]. Myoclonus presents more frequently in atypical forms of AD with major neocortical affectation [15,16], as well as in other atypical forms of AD with other dementia symptoms and parkinsonism [17,18,19,20]. In addition, relative seizure rates increase with earlier age-at-onset in AD, FTD, and other dementias, and relative myoclonus rates increase with earlier age-at-onset in all groups [11,21,22,23,24,25]. In the general population, an increase in age-related seizures due to vascular disease or other morbidities has also been observed [2]. This fact contradicts with the increased risk of seizures in early-onset AD, which could be related to the existence of risk factors associated with the altered expression of genes that regulate the activity of neural networks in young patients with AD [26,27,28,29,30,31,32].

MAPT-related disorders present heterogeneous clinical manifestations. FTDP-17, PSP, corticobasal degeneration (CBD), mild late-onset parkinsonism, and dementia with epilepsy are the most commonly MAPT associated disease [33,34]. In a recent report, a case-control study using whole-exome sequencing data from 522 early-onset cases and 584 controls showed that the 17q21.31 MAPT duplication causes early-onset dementia with AD clinical phenotype without deposits of the amyloid-β peptide [35]. However, mutations or polymorphisms in MAPT have not been identified in patients with AD. In addition, typical familial forms of AD are due to autosomal dominant mutations present in the amyloid precursor protein (APP) gene, the precursor to the amyloid- β peptide, in the presenilin 1 (*PSEN1*) or 2 (*PSEN2*) genes, or to duplications of APP. These mutations give rise to early-onset forms of the disease [36,37] (Alzheimer Disease & Frontotemporal Dementia Mutation Database, Available online: http://www.molgen.ua.ac.be/ADMutations). Accordingly, mutations found in AD patients displaying epileptic seizures are related to the APP processing and the formation of the amyloid-β peptide. Thus, in patients with early-onset familial AD, the risk of epileptic seizures is significantly higher in patients presenting certain mutations and duplications of APP [38] or mutations in the *PSEN1* [39] and, to a lesser extend in patients with mutations in *PSEN2* [29,30,31,32,38,39,40,41]. On the other hand, apolipoprotein E4 allele (apoE4) is the main genetic risk factor for late-onset AD [42] and it confers a greater AD risk with earlier age of onset (reviewed in [43]). Likewise, apoE4 allele inheritance has been related to increased risk of late posttraumatic seizures in patients [44]. 

However, statistical frequencies of epileptic seizures or myoclonus in populations of people with dementia are difficult to estimate, partly because the proportion of patients with dementia varies from 3.1 to 29.1%, depending on the diagnostic criteria commonly applied to this population [45], and also because the detection of non-convulsive seizures, that may be frequent in AD patients, is difficult with the habitual methodological systems [46,47]. Moreover, the information on epidemiologic data from patients with dementia suffering epilepsy and from cases of epileptic patients that develop dementia is, so far, insufficient [48,49].

## 2. AD and Seizures

AD is the most common cause of dementia and it is also the most common neurodegenerative disorder, presenting progressive loss of neurons and synapsis that causes cognitive impairment and ataxia, and leads to severe incapacity and death. The neuropathological hallmarks of the disease are the intraneuronal neurofibrillary tangles (NFTs) composed of hyperphosphorylated tau [50,51,52,53,54,55], and the senile plaques, extracellular deposits of amyloid-β filaments surrounded by altered glia, dystrophic axons and dendritic processes [37,56] (Table 1). Both senile plaques and NFTs anomalies cause the activation of microglia and astrocytes, and they are particularly abundant in regions significantly affected by neuronal death and loss of synapses. In the early stages of AD, NFTs, amyloid-β deposits and dystrophic neurites are mainly found in the hippocampus and the entorhinal cortex, key brain regions for learning and memory [57,58]. These cortical networks are among the most epileptogenic formations of the brain and are also tightly involved in temporal lobe epilepsy (TLE) [59]. Moreover, significant impairments in synapsis and functional neuronal networks have been detected in both, AD and TLE [25,60]. The hippocampus of patients with AD and epilepsy shows similar pathologies, like circuitry reorganization and loss of hippocampal neurons [61]. Altered synaptic function is also common to the pathogenesis of both AD and epilepsy [25], as will be mentioned later.

It has been suggested that increased abnormal tau and amyloid-β proteins in mouse models, as occurs in AD, may present a synergic effect on the generation of epileptic seizures [62,63,64]. Abnormal amyloid-β protein overexpression in animal models has been shown to produce hyperexcitability of hippocampal neurons, an event that is then followed by compensatory remodeling of inhibitory mechanisms [63]. Other authors have suggested that increased abnormal amyloid-β peptide produces aberrant excitatory activity that directly results in epileptic seizures [65]. However, these effects of amyloid-β peptide overexpression did not take place in the absence of normal tau protein [66,67,68]. Hence, the precise cause of the generation of epileptic seizures in patients with AD has not been fully defined, although they may be the result of circuitry hyperexcitability produced by alterations in the excitatory-inhibitory systems, as occurs in animal models [63,69]. On the other hand, it has also been postulated that abnormal patterns of neuronal activity in brain may also trigger and perpetuate synaptic mechanisms of neurodegeneration [70,71]. 

Hyperphosphorylated tau aggregates have been observed in patients with epilepsy [72,73,74,75,76], as well as in different models of chemically and electrically generated epilepsy [77,78,79,80], although tau hyperphosphorylation is not always associated with seizures. Aggregates of hyperphosphorylated tau in the brain of patients with epilepsy were recognized by phospho-S396 [75], AT8 (against phospho-S202, -T205, -S199 and -S208 tau epitopes) [72,73,74,75,76] and AT100 (against phospho-S121 and -T214) [75] antibodies. In TLE, increased tau pathology has been related to epilepsy and cognitive decline [74,75]. Disorders associated with abnormal tau aggregation are generally known as tauopathies. In tauopathies, the accumulation of hyperphosphorylated tau may result from a decreased efficiency of phosphatases, mainly protein phosphatase 2A (PP2A), an increased activation of kinases or both, giving rise to the formation of NFTs [81]. Cyclin dependent kinase 5 (CDK5) and glycogen synthase kinase 3 beta (GSK3β) are among the most relevant protein kinases involved in tau hyperphosphorylation [82]. Additionally, human tau protein-mediated toxicity involves the activities of both GSK3 and Cdk5 kinases [83]. In patients with TLE, an increased expression of CDK5 has been reported, together with an overactivation of GSK-3β [60]. Thus, it seems likely that both kinases may be involved in the hyperphosphorylation of tau in epilepsy. Tau binds to tubulin to stabilize microtubules, and this association is regulated by phosphorylation [84,85,86]. Thus, hyperphosphorylation of tau decreases its affinity to bind microtubules, causing its self-aggregation into neurotoxic paired helical filaments, the principal fibrous structures of the NFTs [87]. Moreover, tubulin detachment of microtubules affects their stability and the regulation of axonal transport [37,88]. Thus, in neurons, tau controls axonal transport and, by binding to other proteins, it also regulates their subcellular localization. In addition, changes in microtubule stability underlay alterations in the localization and organization of other subcellular organelles such as mitochondria [89,90] or lysosomes [91]. Futhermore, it has been recently reported that microtubule destabilization directly affects neuronal network connectivity [92]. A recent review by I. Sotiropoulos et al. in 2017 [93] describes tau atypical functions and additional roles beyond its standard function as microtubule regulating protein.

Multiple neurotransmitter systems are affected in AD with a pattern that correlates with the presence of neuropathological events. Thus, GABAergic, cholinergic and glutamatergic neurotransmission systems are severely impaired [94,95,96] while loss of noradrenergic [61] and serotonergic neurons, as well as neurons producing somatostatin [97], corticotrophin-releasing factor, substance P and neuropeptide Y has also been described [98] (reviewed in [99]). On the other hand, the expression of acetylcholinesterase in AD can be controlled by the presence of both the amyloid-β peptide and the abnormally hyperphosphorylated tau protein (reviewed in [100]). A role of the noradrenergic neuronal system has been suggested as a common feature among AD, Parkinson’s disease, and epilepsy [61].

Synapse and synaptic protein loss is a universal element in the pathologic changes associated with dementia, and correlates with the severity of dementia [101]). It has been reported that dysregulated endocytosis of synaptic AMPA (α-amino-3-hydroxy-5-methyl-4-isoxazolepropionic acid) and NMDA (*N*-methyl-d-aspartateNOT necessary) receptors in AD may contribute to the progressive loss of memory [102] (reviewed by Tang, 2009 [103] and Gu et al., 2009 [104]). Abnormal synaptic AMPA receptors, long-term potentiation and long-term depression have also been described in patients with AD [105]. On the other hand, it has been shown that epilepsy causes a loss of the glutamate receptor subunit GluA1-containing AMPA receptors across the brain [106]. Hyperphosphorylated tau in patients with AD has also been described to alter the normal synaptic function [107], although the way in which tau regulates excitotoxic damage at the molecular level is unknown. In the dendrites, tau influences NMDA receptor-mediated excitotoxicity [108], while a key role for the tau protein in amyloid-β toxicity has been reported by influencing excitotoxicity mediated by the NMDA receptor [108,109,110].

It is well documented that amyloid alters calcium homeostasis in neurons and glia [111,112,113,114] (reviewed in [115]). It has also been reported that the amyloid-β peptide induces a decline in the activity of sodium channels that further alters the activity of parvalbumin positive interneurons and leads to the generation of epileptic seizures [116]. In addition, amyloid-β oligomers might lead to pre- and postsynaptic alterations causing a synaptic damage and impaired synaptic plasticity [117,118], altered hippocampal LTD (Long-term depression) [119], and changes of coordinated network activity [120,121].

It has been suggested that seizure activity in AD may reflect altered neuronal network activity and may contribute to the progression of cognitive impairment [122]. Some cases of patients presenting spontaneous seizures in the early stages of dementia have also been reported, meanwhile cognitive alterations are still minimal and brain images are roughly normal [23,121]. Moreover, new evidence indicates that in the early stages of AD, seizures and network hyperexcitability may appear and contribute to the development of early-onset AD by accelerating the development of cognitive impairment [24,121,122]. The use of various antiepileptic drugs in the prevention of cognitive deterioration in patients [123,124,125] and animal models with AD [23,126] also supports this argument, although data in patients are limited. A case-control study of patients with seizures and AD that were treated with levetiracetam, lamotrigine, or phenobarbital showed that levetiracetam caused fewer adverse events than the other antiepileptic drugs while phenobarbital produced persistent negative cognitive side effects [124]. Moreover, they observed that levetiracetam was associated with improved cognitive performance, specifically attention level and oral fluency items, and that lamotrigine had a better effect on mood. However, there were no significant differences in seizure reduction efficacy among the three drugs [124] (reviewed in [80]). Additionally, non-convulsive seizures and epileptiform activity have been reported in patients with AD [24,127,128]. Subclinical epileptiform activity was detected more than four times in patients with AD than in healthy controls (reviewed in [24]). Patients with AD and subclinical seizures showed faster deterioration of executive function and of cognition than those patients with AD and without epileptic activity. Therefore, precise identification and treatment of epilepsy in such patients may improve their clinical course [23,24]. During sleep, seizures with no overt clinical symptoms have also been observed in patients with AD undergoing EEG (Electroencephalogram) [129]. Sleep is critical for regulation of synaptic efficacy, memory, and learning [130] and those mechanisms of memory consolidation could be altered by seizures and epileptic activity. Therefore, the early development of subclinical hyperexcitability could also contribute to the pathogenesis of AD.

Currently, increasing data from human and animal models point to the importance of different pathophysiological events that take place in both, the generation of epileptic seizures, and the appearance of dementia, such as altered cytoskeletal function leading to changes in neuronal structure and impaired neurotransmitter systems [131], increased amyloid-β protein expression, cerebrovascular alterations, synaptic depression and neuronal hypersynchronization, oxidative stress or neurotrophic factor signaling [21,71,132,133]. Recently, Garg et al., (2018) [134] propose potential shared mechanisms for the pathogenesis of AD and epilepsy, and discuss the novel targets arising from them. These putative targets include neuroinflammation and oxidative stress, tau hyperphosphorylation, and different enzymes that are abnormally expressed in neurodegenerative diseases and epilepsy, such as GSK-3β, PP2A, PKC (Protein kinase C), MMP (Matrix Metalloproteinase) and caspases [134]. 

## 3. Seizures in FTDP-17 and Other Dementias

As mentioned above, compared to the general population, the incidence of seizures and myoclonus is higher in AD [3,4,5,6,7,8,9,10], as well as in other non-AD dementias, such as FTD, PSP, DLB and DS [8,11]. NFTs are found in patients with apparently distinct neurodegenerative diseases and in normal aged brain. In patients with those forms of dementia presenting epileptic seizures, NFTs are invariably observed in brain [135,136], although amyloid-β aggregates have only been described in AD, DLB and DS [137,138] (Table 1).

### 3.1. FTDP-17 and Seizures

**Figure 1:**
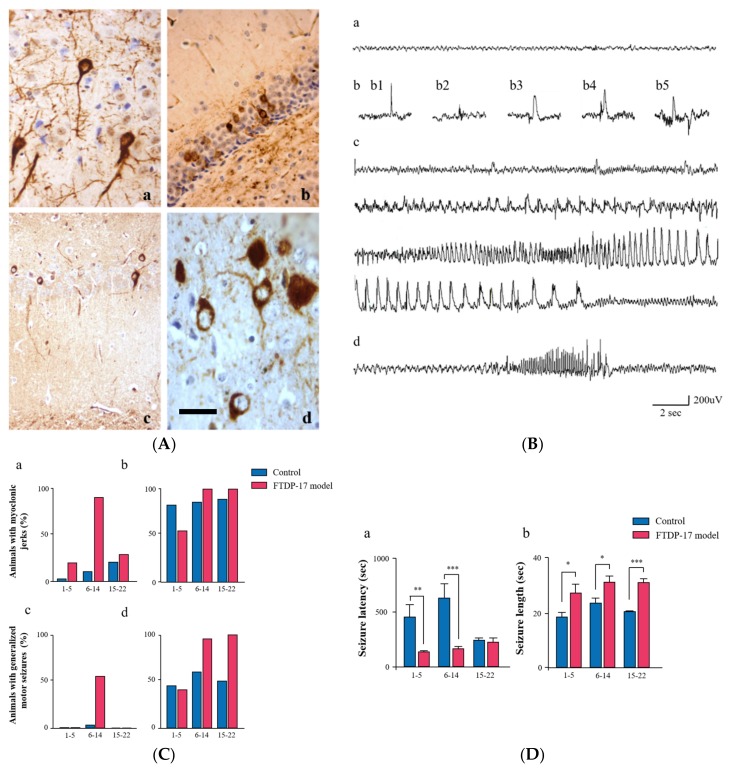
The expression of human TauVLW transgene in FTDP-17 mouse model causes many of the neurological and behavioral abnormalities found in patients with FTDP-17. (**A**) Examples of hyperphosphorylated tau aggregates in brain of FTDP-17 mice at 18 months of age. AT8 tau aggregates in neurons of (a) the CA3 and (c) the CA1 region of the hippocampus; PHF1 tau aggregates in (b) dentate gyrus and (d) amygdala (scale bar corresponds to 32 um in (a), 52 um in (b, c) and 28 um in (d)). (**B**) FTDP-17 mice show epileptic activity. (a) Intracranial record of background activity in control mice; (b) interictal activity, (b1) spike, (b2) polyspike, (b3,4) spike and wave and (b5) polyspike and wave complexes. (**C**) FTDP-17 mice have a higher sensitivity to the PTZ epileptogenic agent. The percentages of mice showing PTZ-induced myoclonic jerks (a and b) and generalized seizures (c and d) are higher in FTDP-17 mice than in controls. (**D**) Seizures induced by PTZ in FTDP-17 mice are more severe than those in control mice. The latency for PTZ-induced seizure onset is shorter (a) and the length of seizures is longer (b) in FTDP-17 mice compared to control mice. * *p* < 0.05; ** *p* < 0.01; *** *p* < 0.001 (*n* = 15–24).

FTDP-17 is an autosomal dominantly inherited neurodegenerative disease, that shows cognitive decline, parkinsonism, and changes in personality and behavior [139,140]. Abnormal forms of hyperphosphorylated tau accumulate in the brain of patients with FTDP-17, in the absence of amyloid-β plaques [139,141] (Table 1). Although tau mutations are not present in the most frequent cases of tauopathies, different mutations in MAPT have been found to cause FTDP-17 and PSP, demonstrating that alterations in tau function result in neurodegeneration and dementia [140,142,143,144,145,146,147]. The P301S MAPT mutation has been associated with FTD and CBD phenotypes, and it has also been linked to an early-onset of rapidly progressive dementia and the presence of myoclonus or seizures [18]. Patients with P301S tau mutation show an extensive filamentous pathology in the brain, consisting of hyperphosphorylated tau in neurons, oligodendroglia and astrocytes. Reactive microglia and neuroimmflamatory mediators have been also described in a case of FTD with P301S mutation [148]. As we will mention below, the mouse transgenic VLW line expressing a 4-repeat tau isoform and bearing three FTDP-17 mutations [149], presents multiple FTDP-17 pathological features [150,151] and shows epileptic activity as well as a higher sensitivity to the GABA_A_ (Gamma aminobutyric acid A) receptor antagonist pentylenetetrazol (PTZ) (Figure 1) [152].

### 3.2. Seizures in PSP

PSP is a neurodegenerative disorder characterized by progressive postural instability, supranuclear ophthalmoplegia, parkinsonism and cognitive decline [153] (reviewed in [154]). Neuropathological diagnosis of PSP is based on the presence of neuronal loss, accumulation of tau protein into NFTs in neurons and astrocytes and dystrophic neurites in basal ganglia and brainstem [155,156,157,158,159] (Table 1). Most frequent cases of PSP are sporadic, although familial cases have also been described [160,161,162,163]. Typical cases of PSP and FTDP-17 can be differentiated by clinical symptoms because dementia appears early in FTDP-17 while it arises late or in benign form in PSP [164,165]. Additionally, atypical familial PSP, often accompanied by parkinsonism, has been related to MAPT mutations [166,167,168,169,170,171]. PSP patients have seizures more frequently than the general population [12,172,173]. On the other hand, epileptic seizures have also been described as a precipitating factor for vascular PSP [174].

### 3.3. Seizures in DLB

DLB is the second most common form of neurodegenerative dementias [175,176]. It presents with psychotic symptoms and cognitive fluctuations. It is characterized by the presence of Lewy bodies, aggregates of alfa-synuclein in neurons [177]. Up to 80% of patients with DLB also show additional AD pathology, amyloid-β plaques, NFTs and neuropil threads [135,178] (Table 1). Moreover, some cases of patients with DLB have been reported to show extensive tauopathy colocalizing with Lewy bodies [179]. Patients with DLB present higher risk to develop seizures and myoclonus, as compared to the general population [11]. Although the presence of EEG abnormalities is valid for a precise diagnosis of DLB, epileptic seizures in patients with erratic cognition may also, at times, produce some symptoms resembling those of DLB [180]. This similarity has questioned the possibilities that DLB was at the origin of epilepsy or that both conditions had an incidental association, particularly in transient epileptic amnesia [181].

### 3.4. Epilepsy in DS

Numerous reports have described a relationship between the presence of AD in Down syndrome (DS) and the generation of epileptic seizures [31,182,183,184,185,186,187,188], correlating with a progressive deterioration of cognitive and motor functions. Both conditions appear in older patients with DS and up to 84% demented patients with DS develop seizures [8]. Patients with early-onset dementia are particularly susceptible to seizures. In addition, late-onset epilepsy in DS is associated with AD, whereas a younger age at onset of epilepsy in DS patients is associated with a lack of dementia [189]. NFTs and senile plaques are found in the brain of patients with DS combined with granulovacuolar degeneration [137,138], although their effects on the generation of epileptic seizures and dementia are also still unknown. A recent review by Zis and Strydom in 2018 [190] reports an overview on the clinical aspects and biomarkers of AD in DS. In this review, authors suggested a correlation between dementia, behavioral alterations and early generation of myoclonus and seizures in DS, and the clinical manifestations of APP mutations with increased amyloid-β (Aβ)42/ Aβ40 ratio [190].

## 4. Animal Models

Multiple experimental models have been generated overexpressing mutant human APP, presenilin and tau proteins, and combinations of these proteins. Convulsive or non-convulsive seizures have been found in almost every AD mouse line, particularly in those transgenic for APP and PSENs. In APP/Aβ transgenic models, high levels of amyloid-β expression were enough to produce epilepsy prior to the appearance of neurodegeneration and neuronal loss, but this effect did not take place in the absence of the wild type tau protein [63,68,108]. Data obtained from those models suggest a key role for the tau protein in amyloid-β toxicity [67,68]. The presence of reactive microglia, astrocytosis, hippocampal synapse loss and impaired synaptic function has been reported in transgenic models of AD and FTDP-17 [191,192]. Some of these factors appeared before fibrillary tau tangles emerged in the P301S FTDP-17 model [191]. In mouse and Drosophila genetic models of epilepsy, tau alters the intrinsic neuronal network excitability in the absence of amyloid-β overexpression [193]. 

As was mentioned above, overexpression of FTDP-17 tau in VLW mice [149] produces distinct pathologies such as hyperphosphorylated tau filaments (Figure 1), lysosomal abnormalities, specific degeneration of the ventral dentate gyrus, and depressive-like behavior [149,150,151,194,195]. Additionally, VLW mice display a notable increase of microglial Iba1+ cells, reactive astrocytes and NFTs in the brain. FTDP-17 human mutant tau overexpression in the VLW model also produces epilepsy and increased PTZ hyperexcitability [152] (Figure 1). Thus, VLW mice present epileptic activity with interictal single spikes, polyspikes, and polyspike and wave complexes corresponding to muscular jerks and generalized seizures. It has also been suggested that network dysfunction in AD is caused by alterations in the GABAergic system [71]. Actually, a relationship between FTDP-17 tau mutations and defects in the GABAergic neurotransmission system has not been described yet, although PTZ hypersensibility in VLW mice suggests the existence of GABAA receptor-mediated hyperexcitability [152]. In addition, reducing tau in AD mouse models prevents excitotoxicity-mediated deficits, while tau-deficient mice showed protection from excitotoxic seizures [62,66,67,68]. In aged mice, tau reduction still conferred resistance to pentylenetetrazole-induced seizures without producing parkinsonian abnormalities in dopamine levels or motor function and did not cause iron accumulation or impaired cognition [196]. 

Data obtained from experimental models of AD, as we mentioned above, suggest a key role for the tau protein in amyloid-β toxicity. EEG analysis of several APP/Aβ transgenic mouse models revealed the presence of cortical and hippocampal synchronous intermittent epileptiform discharges and non-convulsive generalized seizures [63]. In the same report, authors propose that those alterations may be caused by an aberrant activity of excitatory neuronal networks which may induce compensatory inhibitor mechanisms in hippocampal circuits. Thus, they suggested that APP/Aβ transgene overexpression could lead to increased synaptic inhibition and loss of synaptic plasticity [63]. Sodium channel Nav1.1 expression is downregulated in GABAergic interneurons in APP/Aβ transgenic models [197]. Consequently, action potential firing is altered in these interneurons [116] similar to what occurs in SCN1A (Sodium channel protein type 1 subunit alpha) mutant models. Seizures in APP and SCN1A mice can potentially be explained by the reduction of GABAergic interneuron excitability and the altered inhibitory control of downstream targets [115]. On the other hand, it has been observed that the modifications that prevent the appearance of epileptic seizures in these APP/Aβ models also prevent the emergence of cognitive deficits, indicating the existence of common features responsible for both processes [71,116]. 

The tau A152T mutation increases risk for tauopathies, including AD and FTD diseases, such as PSP and CBD [198,199,200,201,202]. Overexpression of the human Tau-A152T in mouse models produces age-dependent neuronal loss, cognitive impairments, and spontaneous non-convulsive epileptiform activity [203]. It also enhances extracellular glutamate, cytotoxicity and progressive neuronal loss in the hippocampus [204]. Network hyperexcitability caused by overexpression of mutant tau transgene may be the result of an increased activity of NMDA receptors. The human TauA152T mutation enhances synaptic transmission and susceptibility for epileptiform activity in hippocampal CA3 area [204] and those effects seem to be caused by an alteration in the proteasome function that delays tau clearance, without alterations in autophagy function [205]. Actually, activation of autophagy in a zebrafish model improved tau clearance and ameliorated its toxicity [205]. 

Hyperphosphorylated tau in NFTs is also found in traumatic brain injury, in long-term and drug resistant epilepsy [72,76]. In experimental models, NFTs are also present in amygdala kindling, post-kainic acid status epilepticus, and posttraumatic epilepsy [206,207]. Therefore, in those animal models it was also suggested that hyperphosphorylated tau could be involved in epileptogenesis. Moreover, PP2A activity is decreased in those models, while treatments with sodium selenate, an activator of PP2A, decreases tau hyperphosphorylation and diminishes epileptogenesis [206,207] (see review in [208]). On the other hand, PTZ effects on tau phosphorylation do not seem to be the direct effect of the presence of epileptic seizures but rather to the alterations caused in glutamate and/or GABAA receptors. Thus, it seems unlikely that hyperphosphorylation of tau was a consequence of epileptic seizures and the way that tau may regulate excitotoxic responses needs to be explored. The VLW model of FTDP-17 presents with cortical and hippocampal alterations [150,151], epilepsy and hyperexcitability [152], resulting from overexpression of FTDP-17 human mutant tau in the absence of Aβ pathology. The anatomical connection between epilepsy and the hippocampal region was first reported in patient H.M., (see review in [209]). Experimental neurosurgery performed in the medial temporal lobe containing the hippocampus controlled his epileptic seizures. The study of H.M. also established key principles about the vulnerability of the hippocampus and its role in epilepsy. As was mentioned above, TLE, the most prevalent form of focal epilepsy, presenting with complex partial seizures, is also associated with neuronal loss and gliosis in the hippocampus. Glial proliferation, particularly astrocytic, is believed to be responsible for the glutamate excess linked to seizure generation in TLE, while cognitive deficits are also present. Moreover, high levels of GFAP have been suggested as being among the earliest and most sensitive features of neuronal toxicity [210]. In this regard, reactive astrogliosis in addition to tau phosphorylation could be potential factors inducing neurodegeneration. Thus, defects in the hippocampal region in VLW mice may make them more vulnerable to epilepsy and to PTZ-induced epilepsy. 

## 5. Conclusions

Epileptic seizures are more common in patients with dementia than in the general elderly population and their appearance has been associated with the progression of cognitive impairment. Both abnormally phosphorylated tau and amyloid-β overexpression in mouse models produce aberrant excitatory activity, although amyloid-β excitotoxicity depends on the presence of tau. On the other hand, hyperphosphorylated tau regulates excitotoxic damage and increases GABAA receptor-mediated hyperexcitability in the absence of abnormal amyloid-β peptide. The connection between abnormally increased network excitability and cognitive deterioration has driven a novel field of research focused on searching for plausible common targets for both conditions. The efficacy of various antiepileptic drugs has been demonstrated in the prevention of cognitive impairment in cases of patients with AD and, most extensively, in experimental models of epilepsy and AD. Moreover, attempts should also be done to evaluate the use of drugs targeting tau hyperphosphorylation and amyloid-β accumulation, as well as microtubule-stabilizing agents, as suitable pharmacological interventions in epileptic disorders. Subsequently, the diagnosis of dementia and epilepsy in early stages of the diseases may be helpful for the initiation of treatments that could prevent the generation of epileptic activity and the progression of cognitive decline. Long-term video-EEG recording should be considered to properly evaluate the presence of epileptiform activity in patients with dementia.

## Figures and Tables

**Table 1:** Neuropathological hallmarks of microtubule associated protein tau gene (MAPT)-related diseases with myoclonus and/or epilepsy.

Diseases	β-Amyloid	Phosopho-tau	α-Synuclein	Epilepsy	Myclonus
Alzhemier disease (AD)	Yes	Yes	No	Yes	Yes
**Other dementias:**					
Dementia with Lewy bodies (LBD)	Yes	Yes	Yes	Yes	Yes
Frontotemporal dementia (FTD)	No	Yes	No	Yes	Yes
Progressive supranuclear palsy (PSP)	No	Yes	No	Yes	Yes
Corticobasal degeneration (CBD)	No	Yes	No	No	Yes
Down síndrome (DS)	Yes	Yes	No	Yes	Yes

## References

[1] Forsgren L., Edvinsson S.O., Blomquist H.K., Heijbel J., Sidenvall R. (1990). Epilepsy in a population of mentally retarded children and adults. Epilepsy Res..

[2] Hesdorffer D.C., Hauser W.A., Annegers J.F., Kokmen E., Rocca W.A. (1996). Dementia and adult-onset unprovoked seizures. Neurology.

[3] Sherzai D., Losey T., Vega S., Sherzai A. (2014). Seizures and dementia in the elderly: Nationwide Inpatient Sample 1999–2008. Epilepsy Behav..

[4] Amatniek J.C., Hauser W.A., DelCastillo-Castaneda C., Jacobs D.M., Marder K., Bell K., Albert M., Brandt J., Stern Y. (2006). Incidence and predictors of seizures in patients with Alzheimer’s disease. Epilepsia.

[5] Mendez M.F., Catanzaro P., Doss R.C., ARguello R., Frey W.H. (1994). Seizures in Alzheimer’s disease: Clinicopathologic study. J. Geriatr. Psychiatry Neurol..

[6] Scarmeas N., Honig L.S., Choi H., Cantero J., Brandt J., Blacker D., Albert M., Amatniek J.C., Marder K., Bell K. (2009). Seizures in Alzheimer disease: Who, when, and how common?. Arch. Neurol..

[7] Romanelli M.F., Morris J.C., Ashkin K., Coben L.A. (1990). Advanced Alzheimer’s disease is a risk factor for late-onset seizures. Arch. Neurol..

[8] Menéndez M. (2005). Down syndrome, Alzheimer’s disease and seizures. Brain Dev..

[9] Irizarry M.C., Jin S., He F., Emond J.A., Raman R., Thomas R.G., Sano M., Quinn J.F., Tariot P.N., Galasko D.R. (2012). Incidence of new-onset seizures in mild to moderate Alzheimer disease. Arch. Neurol..

[10] Nicastro N., Assal F., Seeck M. (2016). From here to epilepsy: The risk of seizure in patients with Alzheimer’s disease. Epileptic Disord..

[11] Beagle A.J., Darwish S.M., Ranasinghe K.G., La A.L., Karageorgiou E., Vossel K.A. (2017). Relative Incidence of Seizures and Myoclonus in Alzheimer’s Disease, Dementia with Lewy Bodies, and Frontotemporal Dementia. J. Alzheimers Dis..

[12] Nygaard T.G., Duvoisin R.C., Manocha M., Chokroverty S. (1989). Seizures in progressive supranuclear palsy. Neurology.

[13] Hauser W.A., Morris M.L., Heston L.L., Anderson V.E. (1986). Seizures and myoclonus in patients with Alzheimer’s disease. Neurology.

[14] Chen J.Y., Stern Y., Sano M., Mayeux R. (1991). Cumulative risks of developing extrapyramidal signs, psychosis, or myoclonus in the course of Alzheimer’s disease. Arch. Neurol..

[15] Alladi S., Xuereb J., Bak T., Nestor P., Knibb J., Patterson K., Hodges J.R. (2007). Focal cortical presentations of Alzheimer’s disease. Brain.

[16] Lee S.E., Rabinovici G.D., Mayo M.C., Wilson S.M., Seeley W.W., DeArmond S.J., Huang E.J., Trojanowski J.Q., Growdon M.E., Jang J.Y. (2011). Clinicopathological correlations in corticobasal degeneration. Ann. Neurol..

[17] Wojcieszek J., Lang A.E., Jankovic J., Greene P., Deck J. (1994). What is it? Case 1, 1994: Rapidly progressive aphasia, apraxia, dementia, myoclonus, and parkinsonism. Mov. Disord..

[18] Bugiani O. (2000). FTDP-17: Phenotypical heterogeneity within P301S. Ann. Neurol..

[19] Sperfeld A.D., Collatz M.B., Baier H., Palmbach M., Storch A., Schwarz J., Tatsch K., Reske S., Joosse M., Heutink P. (1999). FTDP-17: An early-onset phenotype with parkinsonism and epileptic seizures caused by a novel mutation. Ann. Neurol..

[20] Tacik P., Sanchez-Contreras M., DeTure M., Murray M.E., Rademakers R., Ross O.A., Wszolek Z.K., Parisi J.E., Knopman D.S., Petersen R.C. (2017). Clinicopathologic heterogeneity in frontotemporal dementia and parkinsonism linked to chromosome 17 (FTDP-17) due to microtubule-associated protein tau (MAPT) p.P301L mutation, including a patient with globular glial tauopathy. Neuropathol. Appl. Neurobiol..

[21] Larner A.J. (2010). Epileptic seizures in AD patients. Neuromol. Med..

[22] Lozsadi D.A., Larner A.J. (2006). Prevalence and causes of seizures at the time of diagnosis of probable Alzheimer’s disease. Dement. Geriatr. Cogn. Disord..

[23] Vossel K.A., Beagle A.J., Rabinovici G.D., Shu H., Lee S.E., Naasan G., Hegde M., Cornes S.B., Henry M.L., Nelson A.B. (2013). Seizures and epileptiform activity in the early stages of Alzheimer disease. JAMA Neurol..

[24] Vossel K.A., Tartaglia M.C., Nygaard H.B., Zeman A.Z., Miller B.L. (2017). Epileptic activity in Alzheimer’s disease: Causes and clinical relevance. Lancet Neurol..

[25] Noebels J. (2011). A perfect storm: Converging paths of epilepsy and Alzheimer’s dementia intersect in the hippocampal formation. Epilepsia.

[26] Ezquerra M., Carnero C., Blesa R., Gelpí J.L., Ballesta F., Oliva R. (1999). A presenilin 1 mutation (Ser169Pro) associated with early-onset AD and myoclonic seizures. Neurology.

[27] Campion D., Brice A., Hannequin D., Tardieu S., Dubois B., Calenda A., Brun E., Penet C., Tayot J., Martinez M. (1995). A large pedigree with early-onset Alzheimer’s disease: Clinical, neuropathologic, and genetic characterization. Neurology.

[28] Filla A., De Michele G., Cocozza S., Patrignani A., Volpe G., Castaldo I., Ruggiero G., Bonavita V., Masters C., Casari G. (2002). Early onset autosomal dominant dementia with ataxia, extrapyramidal features, and epilepsy. Neurology.

[29] Lindquist S.G., Nielsen J.E., Stokholm J., Schwartz M., Batbayli M., Ballegaard M., Erdal J., Krabbe K., Waldemar G. (2008). Atypical early-onset Alzheimer’s disease caused by the Iranian APP mutation. J. Neurol. Sci..

[30] Larner A.J., Doran M. (2006). Clinical phenotypic heterogeneity of Alzheimer’s disease associated with mutations of the presenilin-1 gene. J. Neurol..

[31] Cabrejo L., Guyant-Maréchal L., Laquerrière A., Vercelletto M., De la Fournière F., Thomas-Antérion C., Verny C., Letournel F., Pasquier F., Vital A. (2006). Phenotype associated with APP duplication in five families. Brain.

[32] Snider B.J., Norton J., Coats M.A., Chakraverty S., Hou C.E., Jervis R., Lendon C.L., Goate A.M., McKeel D.W., Morris J.C. (2005). Novel presenilin 1 mutation (S170F) causing Alzheimer disease with Lewy bodies in the third decade of life. Arch. Neurol..

[33] Van Swieten J.C., Stevens M., Rosso S.M., Rizzu P., Joosse M., de Koning I., Kamphorst W., Ravid R., Spillantini M.G., Niermeijer M.F. (1999). Phenotypic variation in hereditary frontotemporal dementia with tau mutations. Ann. Neurol..

[34] Morris M., Maeda S., Vossel K., Mucke L. (2011). The many faces of tau. Neuron.

[35] Le Guennec K., Quenez O., Nicolas G., Wallon D., Rousseau S., Richard A.C., Alexander J., Paschou P., Charbonnier C., Bellenguez C. (2017). 17q21.31 duplication causes prominent tau-related dementia with increased MAPT expression. Mol. Psychiatry.

[36] Schellenberg G.D., Montine T.J. (2012). The genetics and neuropathology of Alzheimer’s disease. Acta Neuropathol..

[37] Selkoe D.J. (1996). Amyloid beta-protein and the genetics of Alzheimer’s disease. J. Biol. Chem..

[38] Zarea A., Charbonnier C., Rovelet-Lecrux A., Nicolas G., Rousseau S., Borden A., Pariente J., Le Ber I., Pasquier F., Formaglio M. (2016). Seizures in dominantly inherited Alzheimer disease. Neurology.

[39] Shea Y.F., Chu L.W., Chan A.O., Ha J., Li Y., Song Y.Q. (2016). A systematic review of familial Alzheimer’s disease: Differences in presentation of clinical features among three mutated genes and potential ethnic differences. J. Formos. Med. Assoc..

[40] Jayadev S., Leverenz J.B., Steinbart E., Stahl J., Klunk W., Yu C.E., Bird T.D. (2010). Alzheimer’s disease phenotypes and genotypes associated with mutations in presenilin 2. Brain.

[41] Ryan N.S., Nicholas J.M., Weston P.S.J., Liang Y., Lashley T., Guerreiro R., Adamson G., Kenny J., Beck J., Chavez-Gutierrez L. (2016). Clinical phenotype and genetic associations in autosomal dominant familial Alzheimer’s disease: A case series. Lancet Neurol..

[42] Corder E.H., Saunders A.M., Strittmatter W.J., Schmechel D.E., Gaskell P.C., Small G.W., Roses A.D., Haines J.L., Pericak-Vance M.A. (1993). Gene dose of apolipoprotein E type 4 allele and the risk of Alzheimer’s disease in late onset families. Science.

[43] Liu C.C., Kanekiyo T., Xu H., Bu G. (2013). Apolipoprotein E and Alzheimer disease: Risk, mechanisms and therapy. Nat. Rev. Neurol..

[44] Diaz-Arrastia R., Gong Y., Fair S., Scott K.D., Garcia M.C., Carlile M.C., Agostini M.A., Van Ness P.C. (2003). Increased risk of late posttraumatic seizures associated with inheritance of APOE epsilon4 allele. Arch. Neurol..

[45] Erkinjuntti T., Ostbye T., Steenhuis R., Hachinski V. (1997). The effect of different diagnostic criteria on the prevalence of dementia. N. Engl. J. Med..

[46] Clemens Z., Janszky J., Szucs A., Békésy M., Clemens B., Halász P. (2003). Interictal epileptic spiking during sleep and wakefulness in mesial temporal lobe epilepsy: A comparative study of scalp and foramen ovale electrodes. Epilepsia.

[47] Nilsson D., Fohlen M., Jalin C., Dorfmuller G., Bulteau C., Delalande O. (2009). Foramen ovale electrodes in the preoperative evaluation of temporal lobe epilepsy in children. Epilepsia.

[48] Friedman D., Honig L.S., Scarmeas N. (2012). Seizures and epilepsy in Alzheimer’s disease. CNS Neurosci. Ther..

[49] Subota A., Pham T., Jetté N., Sauro K., Lorenzetti D., Holroyd-Leduc J. (2017). The association between dementia and epilepsy: A systematic review and meta-analysis. Epilepsia.

[50] Montejo de Garcini E., Serrano L., Avila J. (1986). Self assembly of microtubule associated protein tau into filaments resembling those found in Alzheimer disease. Biochem. Biophys. Res. Commun..

[51] Grundke-Iqbal I., Iqbal K., Quinlan M., Tung Y.C., Zaidi M.S., Wisniewski H.M. (1986). Microtubule-associated protein tau. A component of Alzheimer paired helical filaments. J. Biol. Chem..

[52] Goedert M., Spillantini M.G., Cairns N.J., Crowther R.A. (1992). Tau proteins of Alzheimer paired helical filaments: Abnormal phosphorylation of all six brain isoforms. Neuron.

[53] Iqbal K., Wiśniewski H.M., Shelanski M.L., Brostoff S., Liwnicz B.H., Terry R.D. (1974). Protein changes in senile dementia. Brain Res..

[54] Iqbal K., Grundke-Iqbal I., Zaidi T., Merz P.A., Wen G.Y., Shaikh S.S., Wisniewski H.M., Alafuzoff I., Winblad B. (1986). Defective brain microtubule assembly in Alzheimer’s disease. Lancet.

[55] Busciglio J., Lorenzo A., Yeh J., Yankner B.A. (1995). beta-amyloid fibrils induce tau phosphorylation and loss of microtubule binding. Neuron.

[56] Masters C.L., Simms G., Weinman N.A., Multhaup G., McDonald B.L., Beyreuther K. (1985). Amyloid plaque core protein in Alzheimer disease and Down syndrome. Proc. Natl. Acad. Sci. USA.

[57] Gómez-Isla T., Price J.L., McKeel D.W., Morris J.C., Growdon J.H., Hyman B.T. (1996). Profound loss of layer II entorhinal cortex neurons occurs in very mild Alzheimer’s disease. J. Neurosci..

[58] Braak H., Braak E. (1991). Neuropathological stageing of Alzheimer-related changes. Acta Neuropathol..

[59] Horváth A., Szűcs A., Barcs G., Noebels J.L., Kamondi A. (2016). Epileptic Seizures in Alzheimer Disease: A Review. Alzheimer Dis. Assoc. Disord..

[60] Liu X., Ou S., Yin M., Xu T., Wang T., Liu Y., Ding X., Yu X., Yuan J., Huang H. (2017). *N*-methyl-d-aspartate receptors mediate epilepsy-induced axonal impairment and tau phosphorylation via activating glycogen synthase kinase-3beta and cyclin-dependent kinase 5. Discov. Med..

[61] Szot P. (2012). Common factors among Alzheimer’s disease, Parkinson’s disease, and epilepsy: Possible role of the noradrenergic nervous system. Epilepsia.

[62] Roberson E.D., Halabisky B., Yoo J.W., Yao J., Chin J., Yan F., Wu T., Hamto P., Devidze N., Yu G.Q. (2011). Amyloid-β/Fyn-induced synaptic, network, and cognitive impairments depend on tau levels in multiple mouse models of Alzheimer’s disease. J. Neurosci..

[63] Palop J.J., Chin J., Roberson E.D., Wang J., Thwin M.T., Bien-Ly N., Yoo J., Ho K.O., Yu G.Q., Kreitzer A. (2007). Aberrant excitatory neuronal activity and compensatory remodeling of inhibitory hippocampal circuits in mouse models of Alzheimer’s disease. Neuron.

[64] Scharfman H.E. (2012). Alzheimer’s disease and epilepsy: Insight from animal models. Future Neurol..

[65] Minkeviciene R., Rheims S., Dobszay M.B., Zilberter M., Hartikainen J., Fülöp L., Penke B., Zilberter Y., Harkany T., Pitkänen A. (2009). Amyloid beta-induced neuronal hyperexcitability triggers progressive epilepsy. J. Neurosci..

[66] Bi M., Gladbach A., van Eersel J., Ittner A., Przybyla M., van Hummel A., Chua S.W., van der Hoven J., Lee W.S., Müller J. (2017). Tau exacerbates excitotoxic brain damage in an animal model of stroke. Nat. Commun..

[67] Ittner A., Chua S.W., Bertz J., Volkerling A., van der Hoven J., Gladbach A., Przybyla M., Bi M., van Hummel A., Stevens C.H. (2016). Site-specific phosphorylation of tau inhibits amyloid-β toxicity in Alzheimer’s mice. Science.

[68] Roberson E.D., Scearce-Levie K., Palop J.J., Yan F., Cheng I.H., Wu T., Gerstein H., Yu G.Q., Mucke L. (2007). Reducing endogenous tau ameliorates amyloid beta-induced deficits in an Alzheimer’s disease mouse model. Science.

[69] Palop J.J., Chin J., Mucke L. (2006). A network dysfunction perspective on neurodegenerative diseases. Nature.

[70] Harris J.A., Devidze N., Verret L., Ho K., Halabisky B., Thwin M.T., Kim D., Hamto P., Lo I., Yu G.Q. (2010). Transsynaptic progression of amyloid-β-induced neuronal dysfunction within the entorhinal-hippocampal network. Neuron.

[71] Palop J.J., Mucke L. (2010). Amyloid-beta-induced neuronal dysfunction in Alzheimer’s disease: From synapses toward neural networks. Nat. Neurosci..

[72] Sen A., Thom M., Martinian L., Harding B., Cross J.H., Nikolic M., Sisodiya S.M. (2007). Pathological tau tangles localize to focal cortical dysplasia in older patients. Epilepsia.

[73] Dyment D.A., Smith A.C., Humphreys P., Schwartzentruber J., Beaulieu C.L., Bulman D.E., Majewski J., Woulfe J., Michaud J., Boycott K.M. (2015). Homozygous nonsense mutation in SYNJ1 associated with intractable epilepsy and tau pathology. Neurobiol. Aging.

[74] Tai X.Y., Koepp M., Duncan J.S., Fox N., Thompson P., Baxendale S., Liu J.Y., Reeves C., Michalak Z., Thom M. (2016). Hyperphosphorylated tau in patients with refractory epilepsy correlates with cognitive decline: A study of temporal lobe resections. Brain.

[75] Liu C., Russin J., Heck C., Kawata K., Adiga R., Yen W., Lambert J., Stear B., Law M., Marquez Y. (2017). Dysregulation of PINCH signaling in mesial temporal epilepsy. J. Clin. Neurosci..

[76] Thom M., Liu J.Y., Thompson P., Phadke R., Narkiewicz M., Martinian L., Marsdon D., Koepp M., Caboclo L., Catarino C.B. (2011). Neurofibrillary tangle pathology and Braak staging in chronic epilepsy in relation to traumatic brain injury and hippocampal sclerosis: A post-mortem study. Brain.

[77] Crespo-Biel N., Canudas A.M., Camins A., Pallàs M. (2007). Kainate induces AKT, ERK and cdk5/GSK3beta pathway deregulation, phosphorylates tau protein in mouse hippocampus. Neurochem. Int..

[78] Liang Z., Liu F., Iqbal K., Grundke-Iqbal I., Gong C.X. (2009). Dysregulation of tau phosphorylation in mouse brain during excitotoxic damage. J. Alzheimers Dis..

[79] Tian F.F., Zeng C., Ma Y.F., Guo T.H., Chen J.M., Chen Y., Cai X.F., Li F.R., Wang X.H., Huang W.J. (2010). Potential roles of Cdk5/p35 and tau protein in hippocampal mossy fiber sprouting in the PTZ kindling model. Clin. Lab..

[80] Liu J., Wang L.N., Wu L.Y., Wang Y.P. (2016). Treatment of epilepsy for people with Alzheimer’s disease. Cochrane Database Syst. Rev..

[81] Lee V.M. (1995). Disruption of the cytoskeleton in Alzheimer’s disease. Curr. Opin. Neurobiol..

[82] Yamaguchi H., Ishiguro K., Uchida T., Takashima A., Lemere C.A., Imahori K. (1996). Preferential labeling of Alzheimer neurofibrillary tangles with antisera for tau protein kinase (TPK) I/glycogen synthase kinase-3 beta and cyclin-dependent kinase 5, a component of TPK II. Acta Neuropathol..

[83] Moreno H., Morfini G., Buitrago L., Ujlaki G., Choi S., Yu E., Moreira J.E., Avila J., Brady S.T., Pant H. (2016). Tau pathology-mediated presynaptic dysfunction. Neuroscience.

[84] Weingarten M.D., Lockwood A.H., Hwo S.Y., Kirschner M.W. (1975). A protein factor essential for microtubule assembly. Proc. Natl. Acad. Sci. USA.

[85] Hirokawa N., Shiomura Y., Okabe S. (1988). Tau proteins: The molecular structure and mode of binding on microtubules. J. Cell Biol..

[86] Lindwall G., Cole R.D. (1984). Phosphorylation affects the ability of tau protein to promote microtubule assembly. J. Biol. Chem..

[87] Hanger D.P., Hughes K., Woodgett J.R., Brion J.P., Anderton B.H. (1992). Glycogen synthase kinase-3 induces Alzheimer’s disease-like phosphorylation of tau: Generation of paired helical filament epitopes and neuronal localisation of the kinase. Neurosci. Lett..

[88] Grundke-Iqbal I., Iqbal K., Tung Y.C., Quinlan M., Wisniewski H.M., Binder L.I. (1986). Abnormal phosphorylation of the microtubule-associated protein tau (tau) in Alzheimer cytoskeletal pathology. Proc. Natl. Acad. Sci. USA.

[89] Nangaku M., Sato-Yoshitake R., Okada Y., Noda Y., Takemura R., Yamazaki H., Hirokawa N. (1994). KIF1B, a novel microtubule plus end-directed monomeric motor protein for transport of mitochondria. Cell.

[90] Tanaka Y., Kanai Y., Okada Y., Nonaka S., Takeda S., Harada A., Hirokawa N. (1998). Targeted disruption of mouse conventional kinesin heavy chain, kif5B, results in abnormal perinuclear clustering of mitochondria. Cell.

[91] Collot M., Louvard D., Singer S.J. (1984). Lysosomes are associated with microtubules and not with intermediate filaments in cultured fibroblasts. Proc. Natl. Acad. Sci. USA.

[92] Verstraelen P., Detrez J.R., Verschuuren M., Kuijlaars J., Nuydens R., Timmermans J.P., De Vos W.H. (2017). Dysregulation of Microtubule Stability Impairs Morphofunctional Connectivity in Primary Neuronal Networks. Front. Cell. Neurosci..

[93] Sotiropoulos I., Galas M.C., Silva J.M., Skoulakis E., Wegmann S., Maina M.B., Blum D., Sayas C.L., Mandelkow E.M., Mandelkow E. (2017). Atypical, non-standard functions of the microtubule associated Tau protein. Acta Neuropathol. Commun..

[94] Davies P., Maloney A.J. (1976). Selective loss of central cholinergic neurons in Alzheimer’s disease. Lancet.

[95] Perry E.K., Tomlinson B.E., Blessed G., Bergmann K., Gibson P.H., Perry R.H. (1978). Correlation of cholinergic abnormalities with senile plaques and mental test scores in senile dementia. Br. Med. J..

[96] Limon A., Reyes-Ruiz J.M., Miledi R. (2012). Loss of functional GABA(A) receptors in the Alzheimer diseased brain. Proc. Natl. Acad. Sci. USA.

[97] Davies P., Katzman R., Terry R.D. (1980). Reduced somatostatin-like immunoreactivity in cerebral cortex from cases of Alzheimer disease and Alzheimer senile dementa. Nature.

[98] Perry E.K., Blessed G., Tomlinson B.E., Perry R.H., Crow T.J., Cross A.J., Dockray G.J., Dimaline R., Arregui A. (1981). Neurochemical activities in human temporal lobe related to aging and Alzheimer-type changes. Neurobiol. Aging.

[99] Strac D.S., Muck-Seler D., Pivac N. (2015). Neurotransmitter measures in the cerebrospinal fluid of patients with Alzheimer’s disease: A review. Psychiatr. Danub..

[100] García-Ayllón M.S., Small D.H., Avila J., Sáez-Valero J. (2011). Revisiting the Role of Acetylcholinesterase in Alzheimer’s Disease: Cross-Talk with P-tau and β-Amyloid. Front. Mol. Neurosci..

[101] DeKosky S.T., Scheff S.W. (1990). Synapse loss in frontal cortex biopsies in Alzheimer’s disease: Correlation with cognitive severity. Ann. Neurol..

[102] Baskys A., Reynolds J.N., Carlen P.L. (1990). NMDA depolarizations and long-term potentiation are reduced in the aged rat neocortex. Brain Res..

[103] Tang B.L. (2009). Neuronal protein trafficking associated with Alzheimer disease: From APP and BACE1 to glutamate receptors. Cell Adh. Migr..

[104] Gu Z., Liu W., Yan Z. (2009). {beta}-Amyloid impairs AMPA receptor trafficking and function by reducing Ca^2+^/calmodulin-dependent protein kinase II synaptic distribution. J. Biol. Chem..

[105] Walsh D.M., Selkoe D.J. (2007). A beta oligomers—A decade of discovery. J. Neurochem..

[106] Grigorenko E., Glazier S., Bell W., Tytell M., Nosel E., Pons T., Deadwyler S.A. (1997). Changes in glutamate receptor subunit composition in hippocampus and cortex in patients with refractory epilepsy. J. Neurol. Sci..

[107] Pooler A.M., Noble W., Hanger D.P. (2014). A role for tau at the synapse in Alzheimer’s disease pathogenesis. Neuropharmacology.

[108] Ittner L.M., Ke Y.D., Delerue F., Bi M., Gladbach A., van Eersel J., Wölfing H., Chieng B.C., Christie M.J., Napier I.A. (2010). Dendritic function of tau mediates amyloid-beta toxicity in Alzheimer’s disease mouse models. Cell.

[109] DeVos S.L., Goncharoff D.K., Chen G., Kebodeaux C.S., Yamada K., Stewart F.R., Schuler D.R., Maloney S.E., Wozniak D.F., Rigo F. (2013). Antisense reduction of tau in adult mice protects against seizures. J. Neurosci..

[110] Gheyara A.L., Ponnusamy R., Djukic B., Craft R.J., Ho K., Guo W., Finucane M.M., Sanchez P.E., Mucke L. (2014). Tau reduction prevents disease in a mouse model of Dravet syndrome. Ann. Neurol..

[111] Dougherty P.J., Davis M.J., Zawieja D.C., Muthuchamy M. (2008). Calcium sensitivity and cooperativity of permeabilized rat mesenteric lymphatics. Am. J. Physiol. Regul. Integr. Comp. Physiol..

[112] Kelly B.L., Ferreira A. (2006). beta-Amyloid-induced dynamin 1 degradation is mediated by *N*-methyl-d-aspartate receptors in hippocampal neurons. J. Biol. Chem..

[113] Hermann D., Mezler M., Müller M.K., Wicke K., Gross G., Draguhn A., Bruehl C., Nimmrich V. (2013). Synthetic Aβ oligomers (Aβ(1-42) globulomer) modulate presynaptic calcium currents: Prevention of Aβ-induced synaptic deficits by calcium channel blockers. Eur. J. Pharmacol..

[114] Ramsden M., Henderson Z., Pearson H.A. (2002). Modulation of Ca^2+^ channel currents in primary cultures of rat cortical neurones by amyloid beta protein (1-40) is dependent on solubility status. Brain Res..

[115] Yu F.H., Mantegazza M., Westenbroek R.E., Robbins C.A., Kalume F., Burton K.A., Spain W.J., McKnight G.S., Scheuer T., Catterall W.A. (2006). Reduced sodium current in GABAergic interneurons in a mouse model of severe myoclonic epilepsy in infancy. Nat. Neurosci..

[116] Verret L., Mann E.O., Hang G.B., Barth A.M., Cobos I., Ho K., Devidze N., Masliah E., Kreitzer A.C., Mody I. (2012). Inhibitory interneuron deficit links altered network activity and cognitive dysfunction in Alzheimer model. Cell.

[117] Nalbantoglu J., Tirado-Santiago G., Lahsaïni A., Poirier J., Goncalves O., Verge G., Momoli F., Welner S.A., Massicotte G., Julien J.P., Shapiro M.L. (1997). Impaired learning and LTP in mice expressing the carboxy terminus of the Alzheimer amyloid precursor protein. Nature.

[118] Shankar G.M., Li S., Mehta T.H., Garcia-Munoz A., Shepardson N.E., Smith I., Brett F.M., Farrell M.A., Rowan M.J., Lemere C.A. (2008). Amyloid-beta protein dimers isolated directly from Alzheimer’s brains impair synaptic plasticity and memory. Nat. Med..

[119] Li S., Hong S., Shepardson N.E., Walsh D.M., Shankar G.M., Selkoe D. (2009). Soluble oligomers of amyloid Beta protein facilitate hippocampal long-term depression by disrupting neuronal glutamate uptake. Neuron.

[120] Nimmrich V., Ebert U. (2009). Is Alzheimer’s disease a result of presynaptic failure? Synaptic dysfunctions induced by oligomeric beta-amyloid. Rev. Neurosci..

[121] Cretin B., Philippi N., Dibitonto L., Blanc F. (2017). Epilepsy at the prodromal stages of neurodegenerative diseases. Geriatr. Psychol. Neuropsychiatr. Vieil..

[122] Leonard A.S., McNamara J.O. (2007). Does epileptiform activity contribute to cognitive impairment in Alzheimer’s disease?. Neuron.

[123] Damar U., Gersner R., Johnstone J.T., Schachter S., Rotenberg A. (2017). Huperzine A: A promising anticonvulsant, disease modifying, and memory enhancing treatment option in Alzheimer’s disease. Med. Hypotheses.

[124] Cumbo E., Ligori L.D. (2010). Levetiracetam, lamotrigine, and phenobarbital in patients with epileptic seizures and Alzheimer’s disease. Epilepsy Behav..

[125] Werhahn K.J., Trinka E., Dobesberger J., Unterberger I., Baum P., Deckert-Schmitz M., Kniess T., Schmitz B., Bernedo V., Ruckes C. (2015). A randomized, double-blind comparison of antiepileptic drug treatment in the elderly with new-onset focal epilepsy. Epilepsia.

[126] Sanchez P.E., Zhu L., Verret L., Vossel K.A., Orr A.G., Cirrito J.R., Devidze N., Ho K., Yu G.Q., Palop J.J. (2012). Levetiracetam suppresses neuronal network dysfunction and reverses synaptic and cognitive deficits in an Alzheimer’s disease model. Proc. Natl. Acad. Sci. USA.

[127] Armon C., Peterson G.W., Liwnicz B.H. (2000). Alzheimer’s disease underlies some cases of complex partial status epilepticus. J. Clin. Neurophysiol..

[128] Rao S.C., Dove G., Cascino G.D., Petersen R.C. (2009). Recurrent seizures in patients with dementia: Frequency, seizure types, and treatment outcome. Epilepsy Behav..

[129] Lam A.D., Deck G., Goldman A., Eskandar E.N., Noebels J., Cole A.J. (2017). Silent hippocampal seizures and spikes identified by foramen ovale electrodes in Alzheimer’s disease. Nat. Med..

[130] Gais S., Born J. (2004). Declarative memory consolidation: Mechanisms acting during human sleep. Learn. Mem..

[131] Whatley V.J., Harris R.A. (1996). The cytoskeleton and neurotransmitter receptors. Int. Rev. Neurobiol..

[132] Gleichmann M., Mattson M.P. (2010). Alzheimer’s disease and neuronal network activity. Neuromol. Med..

[133] Rothman S.M., Mattson M.P. (2010). Adverse stress, hippocampal networks, and Alzheimer’s disease. Neuromol. Med..

[134] Garg N., Joshi R., Medhi B. (2018). Cracking novel shared targets between epilepsy and Alzheimer’s disease: Need of the hour. Rev. Neurosci..

[135] Joachim C.L., Morris J.H., Kosik K.S., Selkoe D.J. (1987). Tau antisera recognize neurofibrillary tangles in a range of neurodegenerative disorders. Ann. Neurol..

[136] Wakabayashi K., Hansen L.A., Vincent I., Mallory M., Masliah E. (1997). Neurofibrillary tangles in the dentate granule cells of patients with Alzheimer’s disease, Lewy body disease and progressive supranuclear palsy. Acta Neuropathol..

[137] Dickson D.W., Ruan D., Crystal H., Mark M.H., Davies P., Kress Y., Yen S.H. (1991). Hippocampal degeneration differentiates diffuse Lewy body disease (DLBD) from Alzheimer’s disease: Light and electron microscopic immunocytochemistry of CA2-3 neurites specific to DLBD. Neurology.

[138] Terry R.D. (1971). Neuronal fibrous protein in human pathology. J. Neuropathol. Exp. Neurol..

[139] Foster N.L., Wilhelmsen K., Sima A.A., Jones M.Z., D’Amato C.J., Gilman S. (1997). Frontotemporal dementia and parkinsonism linked to chromosome 17: A consensus conference. Conference Participants. Ann. Neurol..

[140] Wszolek Z.K., Tsuboi Y., Ghetti B., Pickering-Brown S., Baba Y., Cheshire W.P. (2006). Frontotemporal dementia and parkinsonism linked to chromosome 17 (FTDP-17). Orphanet J. Rare Dis..

[141] Goedert M., Ghetti B., Spillantini M.G. (2012). Frontotemporal dementia: Implications for understanding Alzheimer disease. Cold Spring Harb. Perspect. Med..

[142] Hutton M., Lendon C.L., Rizzu P., Baker M., Froelich S., Houlden H., Pickering-Brown S., Chakraverty S., Isaacs A., Grover A. (1998). Association of missense and 5′-splice-site mutations in tau with the inherited dementia FTDP-17. Nature.

[143] Spillantini M.G., Bird T.D., Ghetti B. (1998). Frontotemporal dementia and Parkinsonism linked to chromosome 17: A new group of tauopathies. Brain Pathol..

[144] Yasuda M., Takamatsu J., D’Souza I., Crowther R.A., Kawamata T., Hasegawa M., Hasegawa H., Spillantini M.G., Tanimukai S., Poorkaj P. (2000). A novel mutation at position +12 in the intron following exon 10 of the tau gene in familial frontotemporal dementia (FTD-Kumamoto). Ann. Neurol..

[145] Spillantini M.G., Van Swieten J.C., Goedert M. (2000). Tau gene mutations in frontotemporal dementia and parkinsonism linked to chromosome 17 (FTDP-17). Neurogenetics.

[146] Pérez M., Lim F., Arrasate M., Avila J. (2000). The FTDP-17-linked mutation R406W abolishes the interaction of phosphorylated tau with microtubules. J. Neurochem..

[147] Probst A., Götz J., Wiederhold K.H., Tolnay M., Mistl C., Jaton A.L., Hong M., Ishihara T., Lee V.M., Trojanowski J.Q. (2000). Axonopathy and amyotrophy in mice transgenic for human four-repeat tau protein. Acta Neuropathol..

[148] Bellucci A., Bugiani O., Ghetti B., Spillantini M.G. (2011). Presence of reactive microglia and neuroinflammatory mediators in a case of frontotemporal dementia with P301S mutation. Neurodegener. Dis..

[149] Lim F., Hernández F., Lucas J.J., Gómez-Ramos P., Morán M.A., Avila J. (2001). FTDP-17 mutations in tau transgenic mice provoke lysosomal abnormalities and Tau filaments in forebrain. Mol. Cell. Neurosci..

[150] Engel T., Lucas J.J., Gómez-Ramos P., Moran M.A., Avila J., Hernández F. (2006). Cooexpression of FTDP-17 tau and GSK-3beta in transgenic mice induce tau polymerization and neurodegeneration. Neurobiol. Aging.

[151] Llorens-Martin M., Hernandez F., Avila J. (2011). Expression of frontotemporal dementia with parkinsonism associated to chromosome 17 tau induces specific degeneration of the ventral dentate gyrus and depressive-like behavior in mice. Neuroscience.

[152] Garcia-Cabrero A.M., Guerrero-Lopez R., Giraldez B.G., Llorens-Martin M., Avila J., Serratosa J.M., Sanchez M.P. (2013). Hyperexcitability and epileptic seizures in a model of frontotemporal dementia. Neurobiol. Dis..

[153] Steele J.C., Richardson J.C., Olszewski J. (1964). Progressive Supranuclear Palsy. A Heterogeneous Degeneration Involving the Brain Stem, Basal Ganglia and Cerebellum with Vertical Gaze and Pseudobulbar Palsy, Nuchal Dystonia and Dementia. Arch. Neurol..

[154] Litvan I., Agid Y., Calne D., Campbell G., Dubois B., Duvoisin R.C., Goetz C.G., Golbe L.I., Grafman J., Growdon J.H. (1996). Clinical research criteria for the diagnosis of progressive supranuclear palsy (Steele-Richardson-Olszewski syndrome): Report of the NINDS-SPSP international workshop. Neurology.

[155] Pérez M., Valpuesta J.M., de Garcini E.M., Quintana C., Arrasate M., López Carrascosa J.L., Rábano A., García de Yébenes J., Avila J. (1998). Ferritin is associated with the aberrant tau filaments present in progressive supranuclear palsy. Am. J. Pathol..

[156] Dickson D.W., Ahmed Z., Algom A.A., Tsuboi Y., Josephs K.A. (2010). Neuropathology of variants of progressive supranuclear palsy. Curr. Opin. Neurol..

[157] Probst A., Langui D., Lautenschlager C., Ulrich J., Brion J.P., Anderton B.H. (1988). Progressive supranuclear palsy: Extensive neuropil threads in addition to neurofibrillary tangles. Very similar antigenicity of subcortical neuronal pathology in progressive supranuclear palsy and Alzheimer’s disease. Acta Neuropathol..

[158] Sanchez M.P., Gonzalo I., Avila J., De Yebenes J.G. (2002). Progressive supranuclear palsy and tau hyperphosphorylation in a patient with a C212Y parkin mutation. J. Alzheimers Dis..

[159] Morales B., Martinez A., Gonzalo I., Vidal L., Ros R., Gomez-Tortosa E., Rabano A., Ampuero I., Sanchez M., Hoenicka J. (2002). Steele-Richardson-Olszewski syndrome in a patient with a single C212Y mutation in the parkin protein. Mov. Disord..

[160] Brown J., Lantos P., Stratton M., Roques P., Rossor M. (1993). Familial progressive supranuclear palsy. J. Neurol. Neurosurg. Psychiatry.

[161] De Yébenes J.G., Sarasa J.L., Daniel S.E., Lees A.J. (1995). Familial progressive supranuclear palsy. Description of a pedigree and review of the literature. Brain.

[162] Rojo A., Pernaute R.S., Fontán A., Ruíz P.G., Honnorat J., Lynch T., Chin S., Gonzalo I., Rábano A., Martínez A. (1999). Clinical genetics of familial progressive supranuclear palsy. Brain.

[163] Höglinger G.U., Melhem N.M., Dickson D.W., Sleiman P.M., Wang L.S., Klei L., Rademakers R., de Silva R., Litvan I., Riley D.E. (2011). Identification of common variants influencing risk of the tauopathy progressive supranuclear palsy. Nat. Genet..

[164] Williams D.R., Lees A.J. (2009). Progressive supranuclear palsy: Clinicopathological concepts and diagnostic challenges. Lancet Neurol..

[165] Fujioka S., Van Gerpen J.A., Uitti R.J., Dickson D.W., Wszolek Z.K. (2014). Familial progressive supranuclear palsy: A literature review. Neurodegener. Dis..

[166] Oliva R., Tolosa E., Ezquerra M., Molinuevo J.L., Valldeoriola F., Burguera J., Calopa M., Villa M., Ballesta F. (1998). Significant changes in the tau A0 and A3 alleles in progressive supranuclear palsy and improved genotyping by silver detection. Arch. Neurol..

[167] Stanford P.M., Halliday G.M., Brooks W.S., Kwok J.B., Storey C.E., Creasey H., Morris J.G., Fulham M.J., Schofield P.R. (2000). Progressive supranuclear palsy pathology caused by a novel silent mutation in exon 10 of the tau gene: Expansion of the disease phenotype caused by tau gene mutations. Brain.

[168] Donker Kaat L., Boon A.J., Azmani A., Kamphorst W., Breteler M.M., Anar B., Heutink P., van Swieten J.C. (2009). Familial aggregation of parkinsonism in progressive supranuclear palsy. Neurology.

[169] Pastor P., Pastor E., Carnero C., Vela R., García T., Amer G., Tolosa E., Oliva R. (2001). Familial atypical progressive supranuclear palsy associated with homozigosity for the delN296 mutation in the tau gene. Ann. Neurol..

[170] Ros R., Thobois S., Streichenberger N., Kopp N., Sanchez M.P., Perez M., Hoenicha J., Avila J., Honnorat J., de Yebenes J.G. (2005). A new mutation of the tau gene, G303V, in early-onset familial progressive supranuclear palsy. Arch. Neurol..

[171] Im S.Y., Kim Y.E., Kim Y.J. (2015). Genetics of Progressive Supranuclear Palsy. J. Mov. Disord..

[172] Kurihara T., Landau W.M., Torack R.M. (1974). Progressive supranuclear palsy with action myoclonus, seizures. Neurology.

[173] Duvoisin R.C., Golbe L.I., Lepore F.E. (1987). Progressive supranuclear palsy. Can. J. Neurol. Sci..

[174] Lanza G., Papotto M., Pennisi G., Bella R., Ferri R. (2014). Epileptic seizure as a precipitating factor of vascular progressive supranuclear palsy: A case report. J. Stroke Cerebrovasc. Dis..

[175] Campbell S., Stephens S., Ballard C. (2001). Dementia with Lewy bodies: Clinical features and treatment. Drugs Aging.

[176] McKeith I.G., Dickson D.W., Lowe J., Emre M., O’Brien J.T., Feldman H., Cummings J., Duda J.E., Lippa C., Perry E.K. (2005). Diagnosis and management of dementia with Lewy bodies: Third report of the DLB Consortium. Neurology.

[177] McKeith I.G., Galasko D., Kosaka K., Perry E.K., Dickson D.W., Hansen L.A., Salmon D.P., Lowe J., Mirra S.S., Byrne E.J. (1996). Consensus guidelines for the clinical and pathologic diagnosis of dementia with Lewy bodies (DLB): Report of the consortium on DLB international workshop. Neurology.

[178] Ishizawa T., Mattila P., Davies P., Wang D., Dickson D.W. (2003). Colocalization of tau and alpha-synuclein epitopes in Lewy bodies. J. Neuropathol. Exp. Neurol..

[179] Clarimón J., Molina-Porcel L., Gómez-Isla T., Blesa R., Guardia-Laguarta C., González-Neira A., Estorch M., Ma Grau J., Barraquer L., Roig C. (2009). Early-onset familial lewy body dementia with extensive tauopathy: A clinical, genetic, and neuropathological study. J. Neuropathol. Exp. Neurol..

[180] Park I.S., Yoo S.W., Lee K.S., Kim J.S. (2014). Epileptic seizure presenting as dementia with Lewy bodies. Gen. Hosp. Psychiatry.

[181] Ukai K., Fujishiro H., Watanabe M., Kosaka K., Ozaki N. (2017). Similarity of symptoms between transient epileptic amnesia and Lewy body disease. Psychogeriatrics.

[182] Gholipour T., Mitchell S., Sarkis R.A., Chemali Z. (2017). The clinical and neurobehavioral course of Down syndrome and dementia with or without new-onset epilepsy. Epilepsy Behav..

[183] D’ORSI G., Specchio L.M., Epilepsy A.S.G.o.S.M. (2014). Progressive myoclonus epilepsy in Down syndrome patients with dementia. J. Neurol..

[184] Lott I.T., Doran E., Nguyen V.Q., Tournay A., Movsesyan N., Gillen D.L. (2012). Down syndrome and dementia: Seizures and cognitive decline. J. Alzheimers Dis..

[185] De Simone R., Puig X.S., Gélisse P., Crespel A., Genton P. (2010). Senile myoclonic epilepsy: Delineation of a common condition associated with Alzheimer’s disease in Down syndrome. Seizure.

[186] Evenhuis H.M. (1990). The natural history of dementia in Down’s syndrome. Arch. Neurol..

[187] Lai F., Williams R.S. (1989). A prospective study of Alzheimer disease in Down syndrome. Arch. Neurol..

[188] Zis P., Strydom A., Buckley D., Adekitan D., McHugh P.C. (2017). Cognitive ability in Down syndrome and its relationship to urinary neopterin, a marker of activated cellular immunity. Neurosci. Lett..

[189] Puri B.K., Ho K.W., Singh I. (2001). Age of seizure onset in adults with Down’s syndrome. Int. J. Clin. Pract..

[190] Zis P., Strydom A. (2018). Clinical aspects and biomarkers of Alzheimer’s disease in Down syndrome. Free Radic. Biol. Med..

[191] Yoshiyama Y., Higuchi M., Zhang B., Huang S.M., Iwata N., Saido T.C., Maeda J., Suhara T., Trojanowski J.Q., Lee V.M. (2007). Synapse loss and microglial activation precede tangles in a P301S tauopathy mouse model. Neuron.

[192] Murakami T., Paitel E., Kawarabayashi T., Ikeda M., Chishti M.A., Janus C., Matsubara E., Sasaki A., Kawarai T., Phinney A.L. (2006). Cortical neuronal and glial pathology in TgTauP301L transgenic mice: Neuronal degeneration, memory disturbance, and phenotypic variation. Am. J. Pathol..

[193] Holth J.K., Bomben V.C., Reed J.G., Inoue T., Younkin L., Younkin S.G., Pautler R.G., Botas J., Noebels J.L. (2013). Tau loss attenuates neuronal network hyperexcitability in mouse and Drosophila genetic models of epilepsy. J. Neurosci..

[194] Guerrero R., Navarro P., Gallego E., Avila J., de Yebenes J.G., Sanchez M.P. (2008). Park2-null/tau transgenic mice reveal a functional relationship between parkin and tau. J. Alzheimers Dis..

[195] Guerrero R., Navarro P., Gallego E., Garcia-Cabrero A.M., Avila J., Sanchez M.P. (2009). Hyperphosphorylated tau aggregates in the cortex and hippocampus of transgenic mice with mutant human FTDP-17 Tau and lacking the PARK2 gene. Acta Neuropathol..

[196] Li Z., Hall A.M., Kelinske M., Roberson E.D. (2014). Seizure resistance without parkinsonism in aged mice after tau reduction. Neurobiol. Aging.

[197] Kim D.Y., Carey B.W., Wang H., Ingano L.A., Binshtok A.M., Wertz M.H., Pettingell W.H., He P., Lee V.M., Woolf C.J. (2007). BACE1 regulates voltage-gated sodium channels and neuronal activity. Nat. Cell Biol..

[198] Lee S.E., Tartaglia M.C., Yener G., Genç S., Seeley W.W., Sanchez-Juan P., Moreno F., Mendez M.F., Klein E., Rademakers R. (2013). Neurodegenerative disease phenotypes in carriers of MAPT p.A152T, a risk factor for frontotemporal dementia spectrum disorders and Alzheimer disease. Alzheimer Dis. Assoc. Disord..

[199] Kovacs G.G., Wöhrer A., Ströbel T., Botond G., Attems J., Budka H. (2011). Unclassifiable tauopathy associated with an A152T variation in MAPT exon 7. Clin. Neuropathol..

[200] Coppola G., Chinnathambi S., Lee J.J., Dombroski B.A., Baker M.C., Soto-Ortolaza A.I., Lee S.E., Klein E., Huang A.Y., Sears R. (2012). Evidence for a role of the rare p.A152T variant in MAPT in increasing the risk for FTD-spectrum and Alzheimer’s diseases. Hum. Mol. Genet..

[201] Jin S.C., Pastor P., Cooper B., Cervantes S., Benitez B.A., Razquin C., Goate A., Cruchaga C., Ibero-American Alzheimer Disease Genetics Group Researchers (2012). Pooled-DNA sequencing identifies novel causative variants in PSEN1, GRN and MAPT in a clinical early-onset and familial Alzheimer’s disease Ibero-American cohort. Alzheimers Res. Ther..

[202] Kara E., Ling H., Pittman A.M., Shaw K., de Silva R., Simone R., Holton J.L., Warren J.D., Rohrer J.D., Xiromerisiou G. (2012). The MAPT p.A152T variant is a risk factor associated with tauopathies with atypical clinical and neuropathological features. Neurobiol. Aging.

[203] Maeda S., Djukic B., Taneja P., Yu G.Q., Lo I., Davis A., Craft R., Guo W., Wang X., Kim D. (2016). Expression of A152T human tau causes age-dependent neuronal dysfunction and loss in transgenic mice. EMBO Rep..

[204] Decker J.M., Krüger L., Sydow A., Dennissen F.J., Siskova Z., Mandelkow E., Mandelkow E.M. (2016). The Tau/A152T mutation, a risk factor for frontotemporal-spectrum disorders, leads to NR2B receptor-mediated excitotoxicity. EMBO Rep..

[205] Lopez A., Lee S.E., Wojta K., Ramos E.M., Klein E., Chen J., Boxer A.L., Gorno-Tempini M.L., Geschwind D.H., Schlotawa L. (2017). A152T tau allele causes neurodegeneration that can be ameliorated in a zebrafish model by autophagy induction. Brain.

[206] Jones N.C., Nguyen T., Corcoran N.M., Velakoulis D., Chen T., Grundy R., O’Brien T.J., Hovens C.M. (2012). Targeting hyperphosphorylated tau with sodium selenate suppresses seizures in rodent models. Neurobiol. Dis..

[207] Shultz S.R., Wright D.K., Zheng P., Stuchbery R., Liu S.J., Sashindranath M., Medcalf R.L., Johnston L.A., Hovens C.M., Jones N.C. (2015). Sodium selenate reduces hyperphosphorylated tau and improves outcomes after traumatic brain injury. Brain.

[208] Zheng P., Shultz S.R., Hovens C.M., Velakoulis D., Jones N.C., O’Brien T.J. (2014). Hyperphosphorylated tau is implicated in acquired epilepsy and neuropsychiatric comorbidities. Mol. Neurobiol..

[209] Squire L.R. (2009). The legacy of patient H.M. for neuroscience. Neuron.

[210] O’Callaghan J.P., Jensen K.F. (1992). Enhanced expression of glial fibrillary acidic protein and the cupric silver degeneration reaction can be used as sensitive and early indicators of neurotoxicity. Neurotoxicology.

